# Telephone instructions improve the quality of bowel preparation for colonoscopy: A meta-analysis of randomized controlled trials

**DOI:** 10.1371/journal.pone.0289063

**Published:** 2023-11-22

**Authors:** Xueqian He, Xiaoju Lei, Jiaqi Li, Peng Li

**Affiliations:** 1 Laboratory Medicine Center, Department of Clinical Laboratory, Zhejiang Provincial People’s Hospital (Affiliated People’s Hospital), Hangzhou Medical College, Hangzhou, Zhejiang, China; 2 Department of Clinical Laboratory, Sir Run Run Shaw Hospital, Zhejiang University School of Medicine, Hangzhou, Zhejiang, China; 3 Endoscopy Center, Nursing Department, Zhejiang Provincial People’s Hospital (Affiliated People’s Hospital), Hangzhou Medical College, Hangzhou, Zhejiang, China; 4 Cancer Center, Department of Gastroenterology, Zhejiang Provincial People’s Hospital (Affiliated People’s Hospital), Hangzhou Medical College, Hangzhou, Zhejiang, China; The University of Hong Kong Li Ka Shing Faculty of Medicine, HONG KONG

## Abstract

**Objective:**

To evaluate the effect of telephone instructions on the quality of bowel preparation in patients undergoing colonoscopy.

**Methods:**

Online English databases (PubMed, Web of Science, Cochrane Library, and Embase) were screened for randomized controlled trials on telephone instructions regarding bowel preparation for colonoscopy from inception to April 15, 2022. After data extraction, the Review Manager software was used for meta-analysis.

**Results:**

Nine randomized controlled trials with 3,836 patients were included in the meta-analysis. The rate of adequate bowel preparation was significantly higher in the telephone group than in the control group. The pooled relative risk (RR) was 1.17 (95% confidence interval [CI]: 1.05–1.30, P < 0.01). The pooled mean difference (MD) for the Boston Bowel Preparation Scale score was 1.32 (95% CI: 0.15–2.49, P < 0.05), and that for the Ottawa Bowel Preparation Scale score was −1.93 (95% CI: −2.35 to −1.51, P < 0.01). The polyp detection rate was significantly higher in the telephone group than in the control group (RR = 1.58, 95% CI: 1.23–2.04, P < 0.01), whereas no significant difference was noted in the adenoma detection rate between the groups (RR = 1.37, 95% CI: 0.97–1.94, P = 0.08).

**Conclusion:**

Telephone instructions for patients undergoing colonoscopy significantly improved the quality of bowel preparation and increased polyp detection rate.

## 1. Introduction

Colorectal cancer is one of the most common malignant tumors [1]. According to GLOBOCAN2020 data, there were approximately 1.88 million new cases of colorectal cancer and approximately 0.92 million deaths worldwide in 2020 [2]. The incidence rate of colorectal cancer ranks third among malignant tumors worldwide, and its mortality rate ranks second [3]. Colonoscopy is the gold standard for screening and diagnosing colorectal cancer; however, the quality of colonoscopy depends on adequate bowel preparation [4]. The process of bowel preparation is relatively complex as it requires patients to have good compliance with dietary restrictions and purgative administration time, administration method, and dosage, which requires adequate education and instructions [5]. Routine education on bowel preparation often relies on written and oral instruction. However, approximately 20–30% of patients undergoing colonoscopy still do not achieve adequate quality of bowel preparation [6]. Therefore, many institutions use various forms of enhanced education for patients undergoing colonoscopy [7]. The use of telephone has spread worldwide and is increasing in patient education and consultation [8]. There are no official recommendation in the guidelines while it is essential to improve the quality of colonoscopy. In this meta-analysis, we aimed to evaluate the effect of telephone instructions on the quality of bowel preparation in patients undergoing colonoscopy.

## 2. Methods

Meta-analysis was performed according to the standards of the Preferred Reporting Items for Systematic Reviews and Meta-Analyses (PRISMA) [9].

### 2.1 Eligibility criteria

Studies that enrolled patients undergoing colonoscopy, were randomized controlled trials, assessed the effect of telephone instructions on bowel preparation for colonoscopy, reported on the rate of adequate bowel preparation, and written in English language were included in the meta-analysis. Non-randomized controlled trials, cohort studies, case-control trials, conference abstracts, comments, and letters were excluded.

### 2.2 Search strategy

Two researchers independently searched online English databases (PubMed, Web of Science, Cochrane Library, and Embase) for studies from inception to April 15, 2022, using the terms “telephone”, “phone”, “call”, “telemedicine”, “telemedical”, “hotline”, “colonoscopy”, “bowel preparation”, and “colon cleansing”. After eliminating duplicates, studies that did not meet the eligibility criteria were removed based on their titles and abstracts. The full text of the remaining studies was downloaded and reviewed and then screened according to the inclusion and exclusion criteria. Finally, all eligible randomized controlled trials were included in the meta-analysis.

### 2.3 Bias evaluation

Two researchers independently conducted the bias evaluation for each included study using the bias risk assessment tool provided by the Cochrane Collaboration Group, and an independent reviewer resolved any discrepancies.

### 2.4 Data extraction

The following data were extracted from each article: author, year of publication, region, study period, colonoscopy indication, bowel preparation regimen, dietary restrictions, standard instruction format, time and content of telephone instructions, sample size, patient age and sex distribution, bowel preparation evaluation scale, and definition of adequate bowel preparation, as well as various clinical outcomes and adverse reactions. The primary outcome was the rate of adequate bowel preparation, and the secondary outcomes were the bowel preparation quality scores, adenoma detection rate (ADR), polyp detection rate (PDR), cecal intubation rate, cecal intubation time, withdrawal time, willingness to repeat bowel preparation, and adverse events.

### 2.5 Statistical analyses

Review Manager software (version 5.3; Cochrane Collaboration, Oxford, England) was used for the meta-analysis. Continuous data were entered as mean and standard deviation, and dichotomous data as the number of events. Heterogeneity between studies was assessed using the *χ*^2^ test (Cochran Q statistic) and quantified using the I^2^ statistic. Significant heterogeneity was indicated by P < 0.1 and/or *I*^2^ > 50%. A sensitivity analysis was performed if significant heterogeneity was noted among the studies. Data were pooled using random-effects models. Continuous data were evaluated using mean difference (MD) and 95% confidence interval (CI), and dichotomous data were evaluated using relative risk (RR) and 95% CI. P < 0.05 was indicative of a significant difference. Funnel plots were constructed to assess the risk of publication bias across the series for the primary outcomes if there were 10 or more studies included in the meta-analysis.

## 3. Results

### 3.1 Literature search and study characteristics

**Fig 1:**
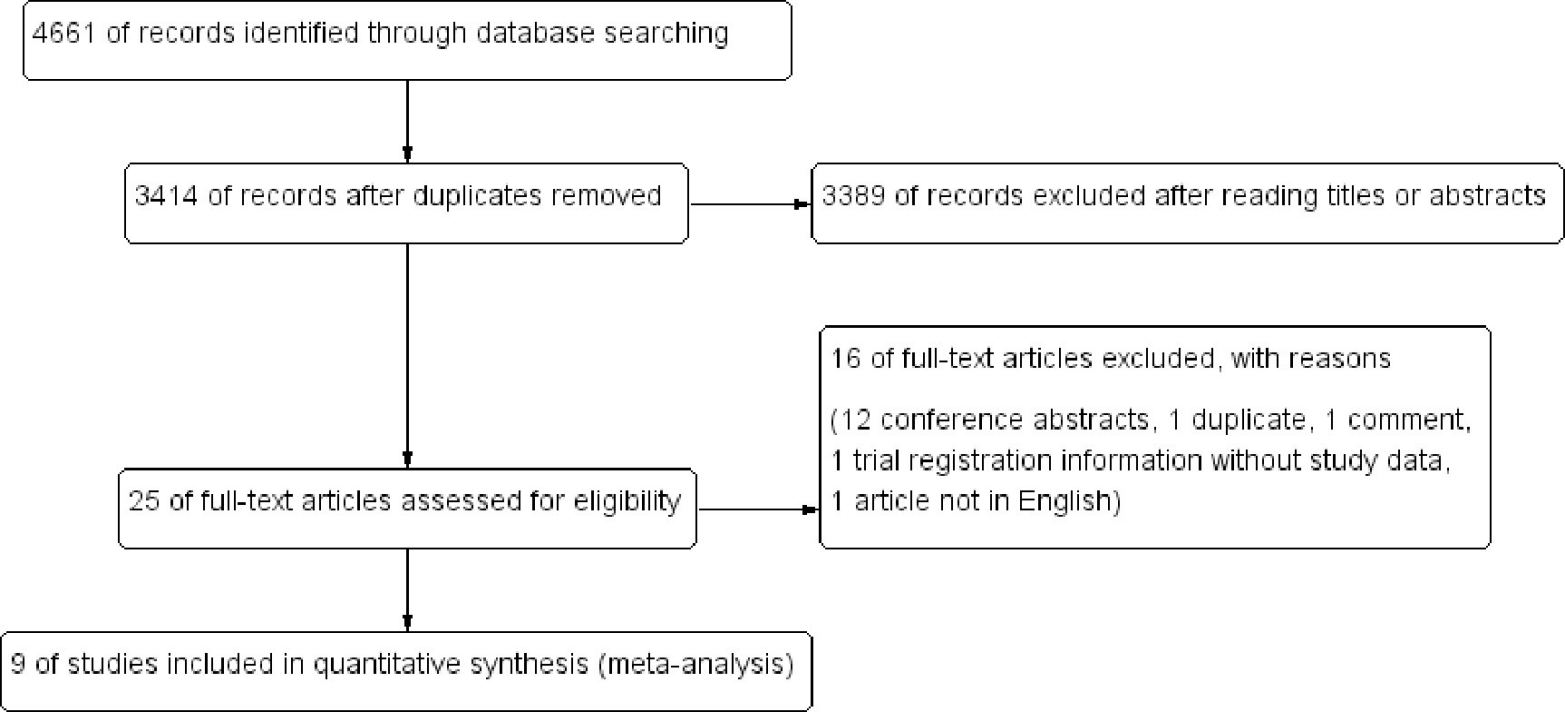
Flow diagram for studies included and excluded.

Using the search strategy, a total of 4,661 articles were initially identified from the online databases. Of these, 1,247 records were removed due to duplications and further 3,389 articles were excluded after reading titles or abstracts. Of the remaining 25 articles, 16 were excluded after reading the full text. Finally, according to the eligibility criteria, 9 randomized controlled trials [10–18] with 3,836 patients were included in the meta-analysis (Fig 1).

**Fig 2:**
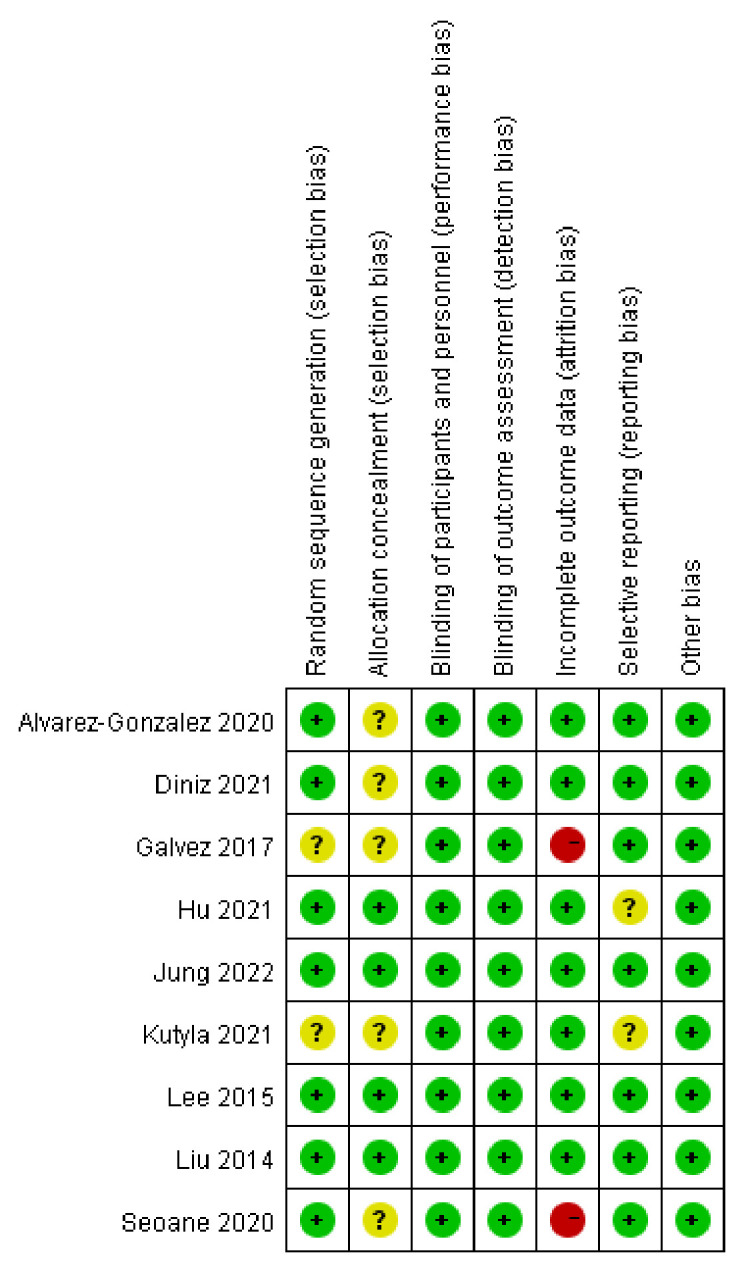
Risk of bias item for each included study.

All included studies were conducted in the past 10 years; two studies were conducted in China [13, 17], two in South Korea [14, 16], two in Spain [10, 18], and one each in Australia [15], Mexico [12], and Brazil [11]. Most patients in the included studies were adults aged >18 years, except for one study [13] that included elderly patients aged >65 years. The purgatives used in most studies were polyethylene glycol (PEG) [10, 12–15, 17, 18] or PEG with ascorbic acid [16, 18], whereas some studies used sodium phosphate [17, 18] or 20% mannitol solution [11]. Five studies [10, 14–16, 18] used a split-dose regimen for bowel preparation, three studies [11, 12, 17] used a single-dose regimen, and the another study [13] adopted an alternative split-dose regimen for patients who were unable to take a single dose of PEG. In seven of the included studies [10–12, 14–16, 18], the Boston Bowel Preparation Scale (BBPS) was used to evaluate the quality of bowel preparation, and adequate bowel preparation was defined as a score of ≥2 in all segments or a total BBPS score of >5. In the remaining two studies [13, 17], the Ottawa Bowel Preparation Scale (OBPS) was used to assess the quality of bowel preparation, where a total score of <6 was considered adequate preparation. The bowel preparation education method in the control groups included written instruction and verbal explanation, whereas patients in the telephone groups received reinforced education via telephone. Most of the included studies [10–14, 16, 17] provided telephone education 1–2 days before colonoscopy, one study [18] provided telephone education 7 days before colonoscopy, and another study [15] provided telephone intervention 2 weeks before colonoscopy. The contents of the telephone intervention included reminders of the appointed colonoscopy, the importance of bowel cleaning, the regimens of bowel preparation, the time of initiating administration, dietary restrictions, and the answers to patients’ questions. Tables 1 and 2 listed characteristics of the included study and patients, while Fig 2 provided the risk of bias item for each included study. The meta-analysis included only 9 studies and it was unnecessary to assess the risk of publication bias by funnel plots.

**Table 1:** Characteristics of included studies.

Study	Country	Study period	Inclusion age criteria	Indication for colonoscopy	Bowel preparation regimen	Standard instructions for bowel preparation	Time for reinforced education by telephone	Scales of bowel preparation quality	Definition of adequate bowel preparation
Alvarez-Gonzalez 2020	Spain	2017.01–2018.06	18–85	surveillance, diagnosis, screening	4L PEG, split dose	written and verbal	24–48 hours before colonoscopy	BBPS	score≥2 in all segments
Diniz 2021	Brazil	2018.02–2018.09	≥18	NA	750 mL 20% mannitol solution, single dose	written and verbal	1 day before colonoscopy	BBPS	score≥2 in all segments
Galvez 2017	Mexico	2016.01–2016.05	≥18	diagnosis, screening	4L PEG, single dose	written and verbal	1 day before colonoscopy	BBPS	total BBPS > 5
Hu 2021	China	2014.12–2015.12	≥65	diagnosis, screening	3-4L PEG, single or split dose	written and verbal	2 days before colonoscopy	OBPS	total OBPS <6
Jung 2022	Korea	2018.09–2020.02	19–75	NA	4L PEG, split dose	written and verbal	8:30 a.m. to 12:00 p.m. on the day before colonoscopy	BBPS	score≥2 in all segments
Kutyla 2021	Australia	2018.01–2018.11	NA	NA	3L PEG, split dose	written	2 weeks prior to their procedure	BBPS	score≥2 in all segments
Lee 2015	Korea	2013.08–2013.12	≥18	screening	2L PEG+ascorbic acid, split dose	written and verbal	between 08:00 and 11:00 2 days before colonoscopy	BBPS	total BBPS≥5
Liu 2014	China	2012.02–2012.07	18–75	surveillance, diagnosis, screening	2L PEG or 1.5L sodium phosphate, single dose	written and verbal	09:00–11:00 on the day before colonoscopy	OBPS	total OBPS <6
Seoane 2020	Spain	2017.11–2018.05	NA	surveillance, diagnosis, screening	4L PEG, 2L PEG +ascorbate, or MCSP, split dose	written and verbal	7 d before the colonoscopy	BBPS	score≥2 in all segments

BBPS, Boston Bowel Preparation Scale; MCSP, magnesium citrate plus sodium picosulfate; NA, not available; OBPS, Ottawa Bowel Preparation Scale; PEG, polyethylene glycol.

**Table 2:** Characteristics of patients from included studies.

Study	Sample size		Average age, yr (mean±SD)		Sex distribution, male (%)	
	Smartphone	Control	Smartphone	Control	Smartphone	Control
Alvarez-Gonzalez 2020	322	329	64.4 (15.7)	63.7 (17.6)	179 (55.6)	185 (56.2)
Diniz 2021	54	55	58±14	57±14	20(37.0)	24(43.6)
Galvez 2017	141	117	51.97±14.78	51.16±15.88	52(36.9)	37(31.6)
Hu 2021	83	79	74.3±7.4	74.7±7.7	42 (50.6)	38 (48.1)
Jung 2022	101	106	56.0±12.4	55.1±10.2	44(43.6)	49(46.2)
Kutyla 2021	53	44	48.3±16	43.1±16	24 (45.3)	20 (45.5)
Lee 2015	126	137	46.0±12.2	47.1±11.8	79 (62.7)	73 (53.3)
Liu 2014	305	300	44.8±12.5	45.7±12.6	160 (52.5)	147 (49.0)
Seoane 2020	738	746	59.1±16.2	59.9±16.0	347 (47.0)	363 (48.7)

SD, standard deviation; NA, not available.

### 3.2 Primary outcome

**Fig 3:**
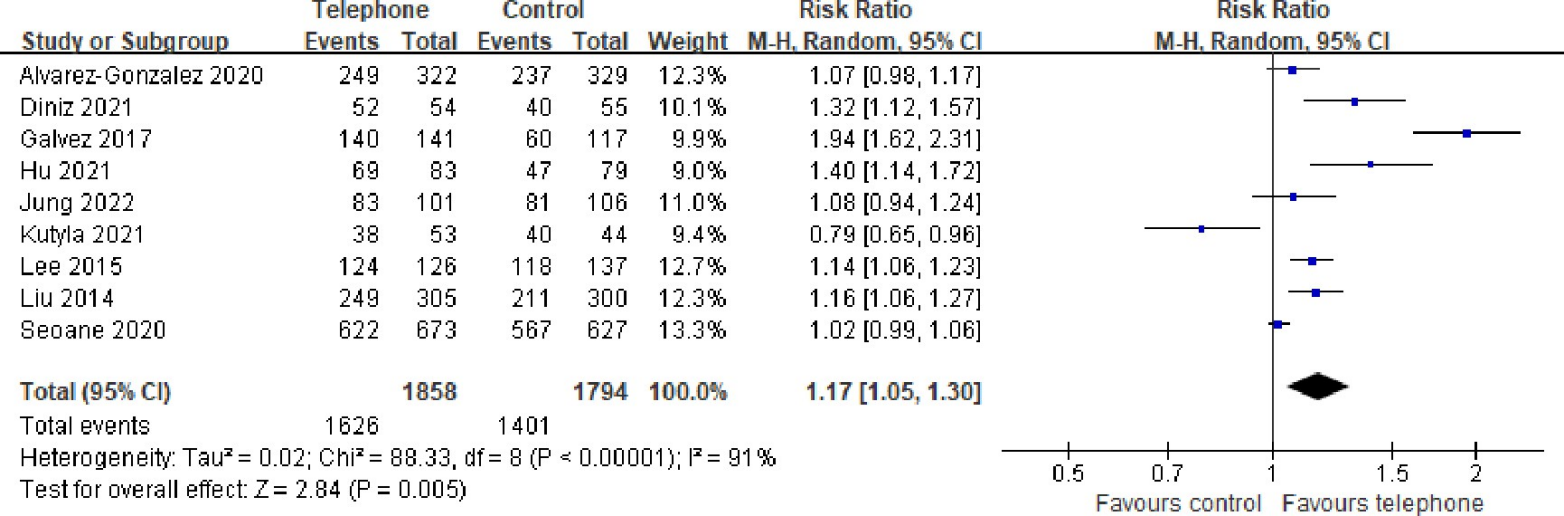
Forest plot comparing rate of adequate bowel preparation.

All of the included studies compared the quality of bowel preparation between the telephone and control groups. The rate of adequate bowel preparation was 87.5% in the telephone group and 78.1% in the control group. The pooled RR of the rate of adequate bowel preparation was 1.17 (95% CI: 1.05–1.30, P < 0.01), which suggested that the rate of adequate bowel preparation in the telephone group was significantly higher than that in the control group (Fig 3). The heterogeneity test showed significant heterogeneity (*I*^2^ = 91%, P < 0.01). A sensitivity analysis was conducted to assess whether any study had a dominant impact on the primary outcomes. The results indicated no significant difference in the RR of the rate of adequate bowel preparation, regardless of whether any of the studies were excluded.

**Fig 4:**
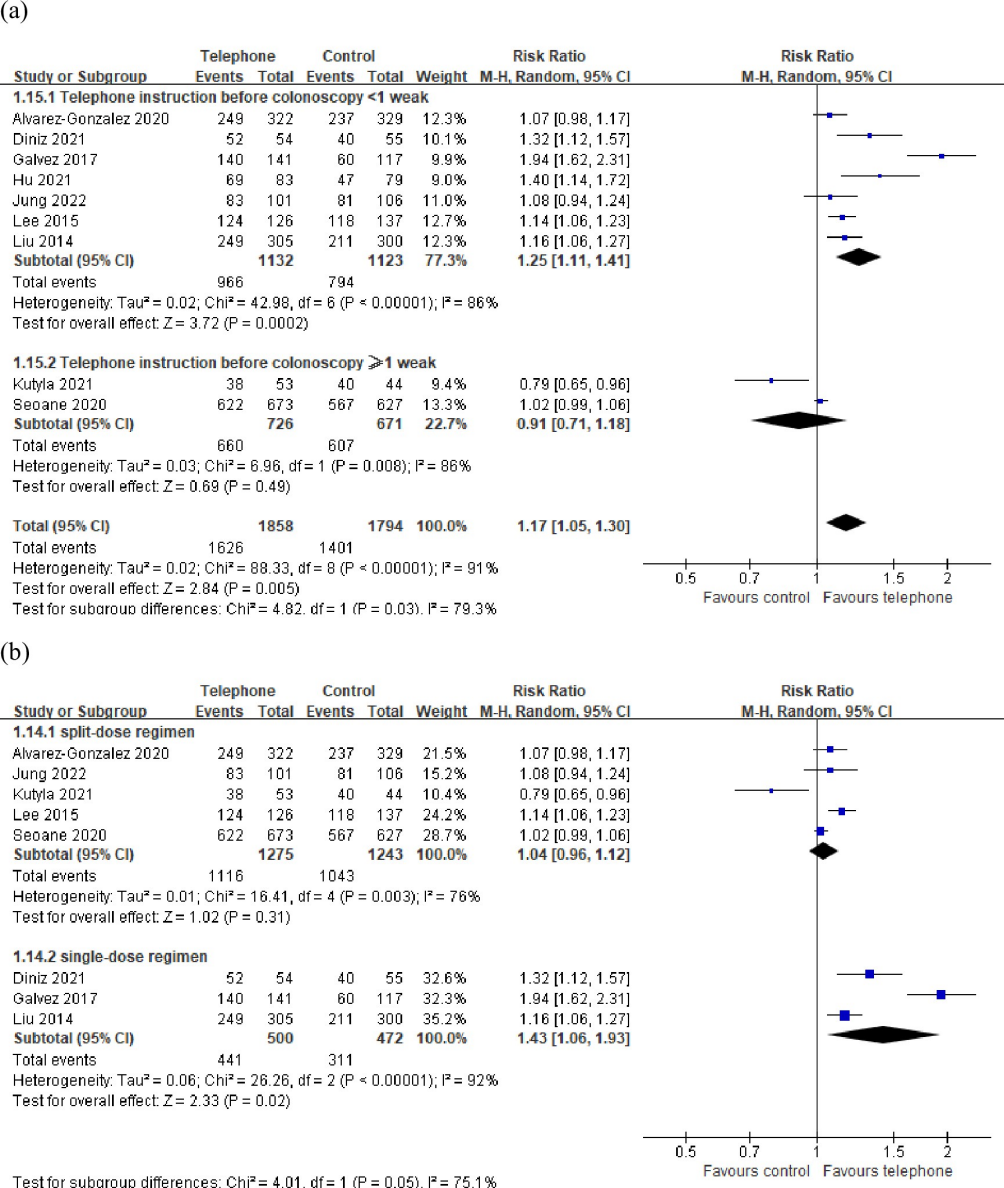
Forest plot forest plots comparing rate of adequate bowel preparation in subgroups of patients. (a) subgroup analysis according to the different time of telephone instruction before the colonoscopy, (b) subgroup analysis according to the different bowel preparation regimens.

The subgroup analysis was conducted according to the different time of telephone instruction before the colonoscopy. There was still significant heterogeneity in the studies included in the <1 week and the ≥ 1 week subgroups. The results of the subgroup analysis showed that the rate of the adequate bowel preparation in the telephone group was significantly higher than that in the control group from the <1 week subgroup (RR = 1.25, 95% CI: 1.11–1.41, P < 0.01, Fig 4A). There was no significant difference in the rate of adequate bowel preparation between the two groups from the ≥ 1 week subgroup (RR = 0.91, 95% CI: 0.71–1.18, P = 0.49, Fig 4A).

The subgroup analysis according to different bowel preparation regimens showed that significant heterogeneity was still existed in the studies included in the split-dose regimen and the single-dose regimen subgroups. The results of the subgroup analysis showed that there was no significant difference in the rate of adequate bowel preparation between the telephone group and the control group from the split-dose regimen subgroup (RR = 1.04, 95% CI: 0.96–1.12, P = 0.31, Fig 4B); the rate of adequate bowel preparation in the smartphone group was significantly higher than that in the control group from the single-dose regimen subgroup (RR = 1.43, 95% CI: 1.06–1.93, P < 0.05, Fig 4B).

### 3.3 Colonoscopy outcomes

**Fig 5:**
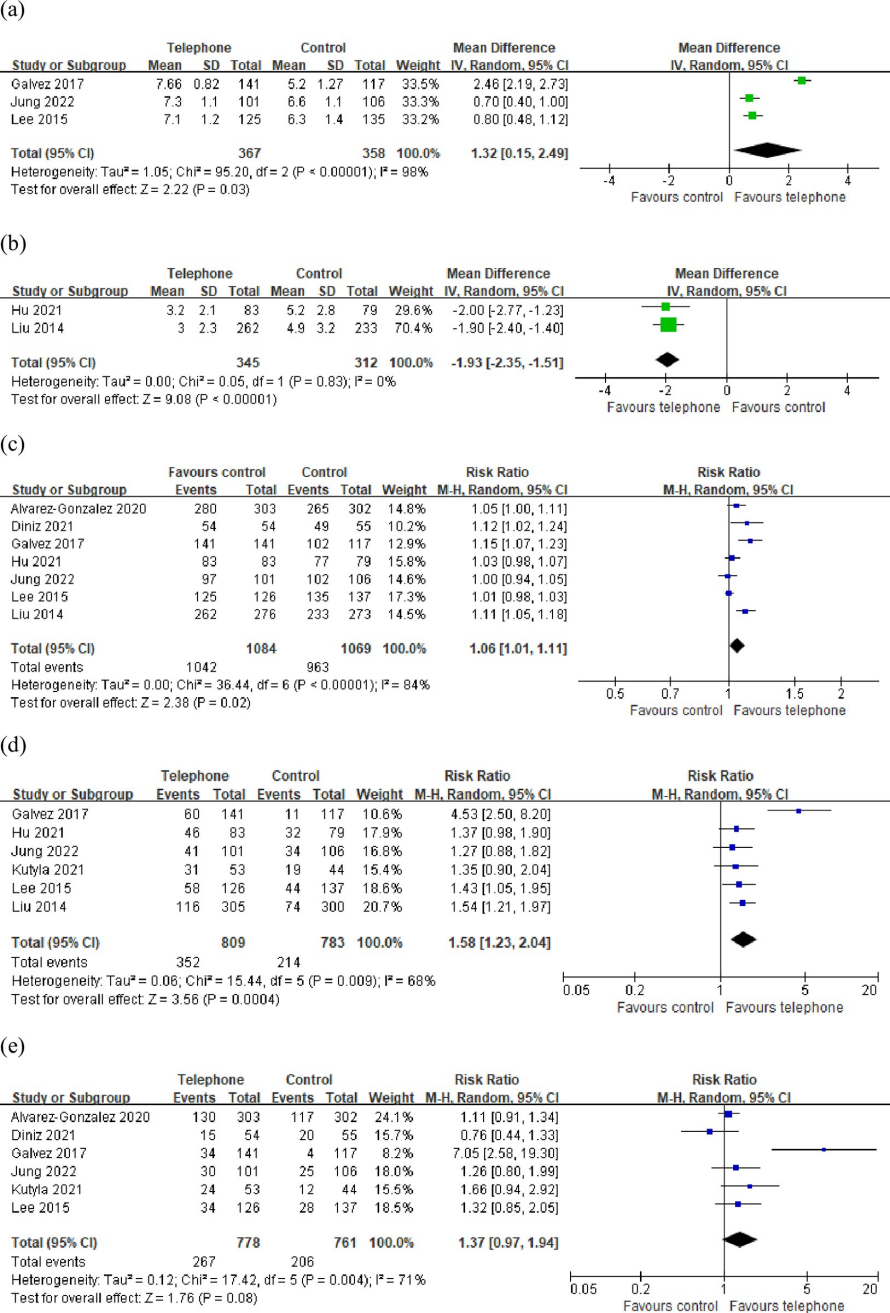
Forest plot comparing (a) total BBPS score, (b) total OBPS score, (c) cecal intubation rate, (d) PDR, (e) ADR.

Three of the included studies [12, 14, 16] evaluated the total BBPS score, and another two [13, 17] evaluated the total OBPS score. The pooled MD for BBPS was 1.32 (95% CI: 0.15–2.49, P < 0.05), and that for OBPS was −1.93 (95% CI: −2.35 to −1.51, P < 0.01). The BBPS score was significantly higher in the telephone group than in the control group (Fig 5A), whereas the OBPS score was significantly lower in the telephone group than in the control group (Fig 5B), both of which suggested that telephone education significantly improved the quality of bowel preparation in patients undergoing colonoscopy. The rate of cecal intubation was significantly higher in the telephone group than in the control group (RR = 1.06, 95% CI: 1.01–1.11, P < 0.05, Fig 5C). PDR was significantly higher in the telephone group than in the control group (RR = 1.58, 95% CI: 1.23–2.04, P < 0.01, Fig 5D); however, there was no significant difference in the ADR between the two groups (RR = 1.37, 95% CI: 0.97–1.94, P = 0.08, Fig 5E). No significant difference was noted in the cecal intubation time between the two groups (P = 0.31); however, the withdrawal time was significantly shorter in the telephone group than in the control group (P < 0.01). No discrepancies were detected in the sensitivity analysis, although there was significant heterogeneity existed in the above results except the OBPS score.

### 3.4 Patient’s experience

**Fig 6:**
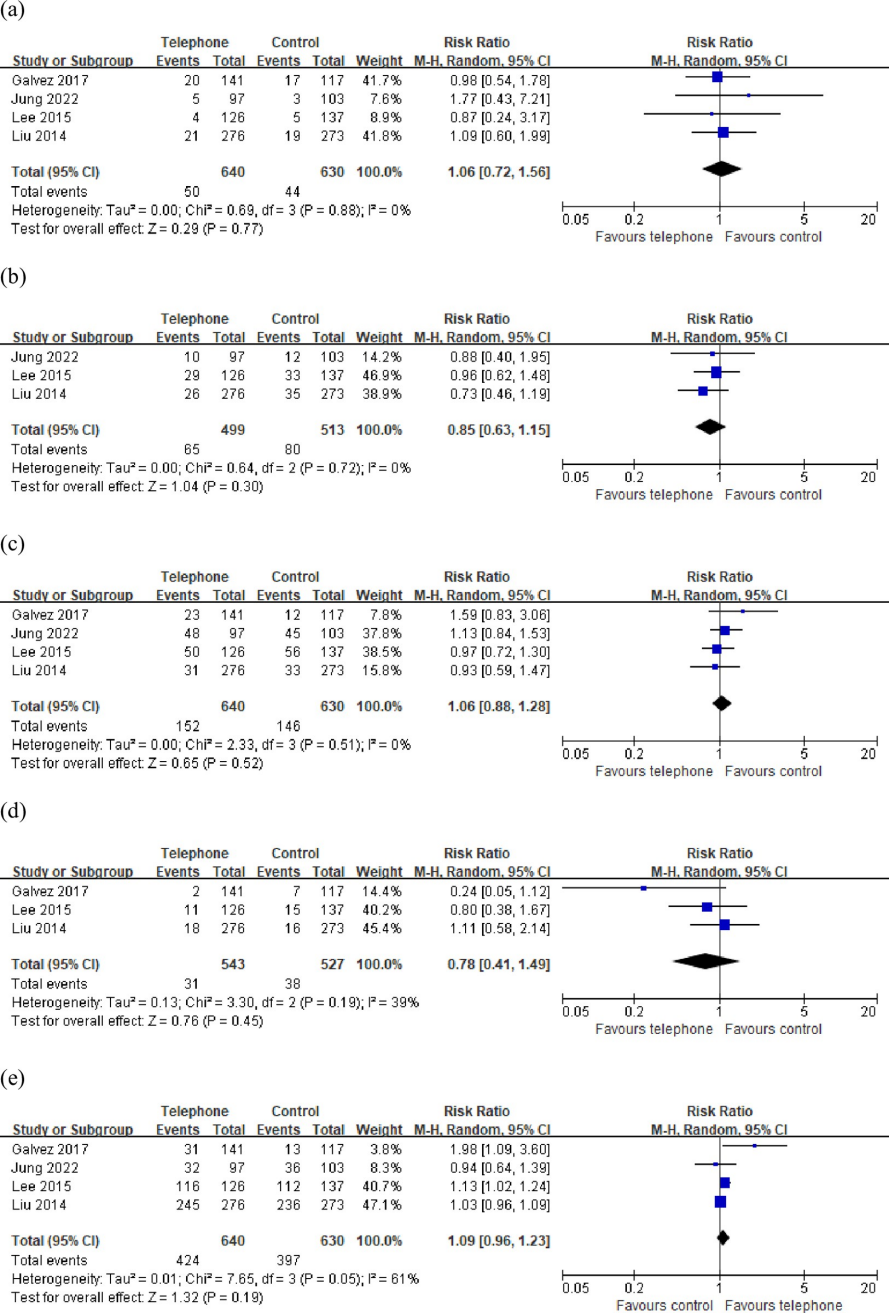
Forest plot concerning (a) patients with abdominal pain, (b) patients with abdominal distension, (c) patients with nausea, (d) patients with vomiting, (e)patient’s willingness to repeat bowel preparation.

No significant difference was noted in the incidence of abdominal pain, abdominal distension, nausea, and vomiting (P = 0.77, 0.30, 0.52, and 0.45, respectively, Fig 6A–6D) without significant heterogeneity between the two groups. The number of patients who were willing to repeat bowel preparation was also not significantly different between the groups (P = 0.19, Fig 6E). The heterogeneity test showed significant heterogeneity. The sensitivity analyses showed no significant difference in the pooled RR was noted with the exclusion of any study.

## 4. Discussion

This meta-analysis included nine randomized controlled trials with 3,836 patients. The results showed that the rate of adequate bowel preparation was 87.5% in the telephone group, which was significantly higher than that in the control group (78.1%). The BBPS score was significantly higher in the telephone group than in the control group, whereas the OBPS score was significantly lower in the telephone group than in the control group, both of which suggested that telephone education significantly improved the quality of bowel preparation in patients undergoing colonoscopy.

Adequate education is the premise to ensure adequate quality of bowel preparation. Before bowel preparation, patients should be actively educated regarding the importance of bowel preparation; time and requirements of dietary restrictions; time, dosage, and method of administration of purgatives; and importance of compliance [19]. At present, many institutions use various forms of reinforced education for appropriate bowel preparation in patients undergoing colonoscopy. A retrospective observational study found enhanced instructions improved the quality of bowel preparation [20]. Several previous meta-analyses of randomized controlled trials showed that enhanced instructions could significantly improve the quality of bowel preparation and ADR [21–25]. These forms of enhanced instructions include visual aids, videos, phones, short message services, social media apps, and smartphones. This study only focused on randomized controlled studies on reinforced education by telephone, which could reduce the heterogeneity caused by different instruction methods. Heterogeneity played an important role in the quality and strength of a meta-analysis. The results showed that telephone instruction could significantly improve the rate of adequate bowel preparation, increase the BBPS scores, and reduce the OBPS scores, all of which suggested that reinforced education via telephone significantly improved the quality of bowel preparation for colonoscopy. At present, telephones have become an indispensable tool in people’s studies, lives, and work and can also be used for medical education and medical follow-up. Since the telephone has widespread use for daily communication, even among the elderly and the low educated, telephoning is a great way to perform reinforced instruction, which has greater advantages than other forms of education. Telephone instructions could remind and emphasize the patients undergoing colonoscopy about the importance of bowel preparation; time and requirements of dietary restrictions; and time, dose, and method of taking purgatives and answer the patients’ questions in a timely manner, which could improve patient compliance and thereby improve the quality of bowel preparation [26]. A prospective cohort study showed that automated time-released colonoscopy preparation reminders to patients via text messages and emails improved bowel preparation quality and resulted in fewer canceled procedures [27]. Childers *et al*. conducted a historically controlled study and found that endoscopy practices may increase revenue, improve scheduling efficiency, and maximize resource utilization by hiring a nurse to make a telephone call to reduce no-shows [28].

The subgroup analysis found that telephone instruction within 1 weak before the colonoscopy improved the quality of bowel preparation, but telephone instruction more than 1 weak before the colonoscopy did not result in a significant improvement in bowel cleansing. Routine oral education and written instruction are easy to forget, which might have affected the effect of education and thus the quality of bowel preparation. Lower levels of education, longer waiting times and noncompliance with instructions were risk factors associated with poor bowel preparation [29]. Since the reminder about bowel preparation may fade over time, enhanced education should be provided in a shorten time before the colonoscopy. Indeed, most of the studies included this meta-analysis instructed patients 1–2 days before endoscopy via telephone [10–14, 16, 17]. The subgroup analysis according to different bowel preparation regimens showed that telephone instruction reached a significant increase in bowel preparation success for the single-dose regimen, but it did not improve preparation quality for the split-dose regimen. Some research studies had demonstrated that a split-dose regimen was as effective as a single dose of purgatives, with better compliance and tolerance [30]. The patients received the single dose regimen might get improvements in compliance and tolerance through telephone instruction, thereby improving the quality of bowel preparation. Future research should evaluate the impact of reinforced instruction on different regimens.

This study has some limitations. First, a pooled analysis of the rate of adequate bowel preparation revealed significant heterogeneity among the studies, despite the sensitivity analysis. Second, bowel preparation purgatives or regimens were different among the included studies, which affected the quality of bowel preparation. Third, the bowel preparation quality assessment scale and definition of adequate bowel preparation were different among the studies, which contributed to the heterogeneity among the studies. In addition, the intervention time and educational content of the telephone were not entirely the same among studies, which also increased the heterogeneity among studies.

## 5. Conclusion

This study suggested that telephone instructions for patients undergoing colonoscopy significantly improved the quality of bowel preparation and increased the PDR.

## Supporting information

S1 Checklist PRISMA 2020 checklist. (DOCX)

S1 File GRADE summary of finding table. (PDF)
