# Supplementary material for: Telephone instructions improve the quality of bowel preparation for colonoscopy: A meta-analysis of randomized controlled trials
Source: PLoS One. 2023 Nov 22;18(11):e0289063. doi: 10.1371/journal.pone.0289063 (PMC10664946; doi:10.1371/journal.pone.0289063)
Supplement: S1 File — (PDF) [file pone.0289063.s002.pdf]

Telephone instructions for colonoscopy bowel preparation

Patient or population: Patients undergoing colonoscopy
Setting: Bowel preparation
Intervention: Telephone instructions
Comparison: Standard instructions

Outcomes	№ of participants (studies) Follow-up	Certainty of the evidence (GRADE)	Relative effect (95% CI)	Anticipated absolute effects	
				Risk with Standard instructions	Risk difference with Telephone instructions
Rate of adequate bowel preparation	3652 (9 RCTs)	⊕⊕◯◯ Low[a,b]	**RR 1.17** (1.05 to 1.30)	781 per 1,000	**133 more per 1,000** (39 more to 234 more)
Total BBPS score	725 (3 RCTs)	⊕⊕◯◯ Low[a,b]	-	The mean total BBPS score was **0**	MD **1.32 higher** (0.15 higher to 2.49 higher)
Total OBPS score	657 (2 RCTs)	⊕⊕⊕⊕ High	-	The mean total OBPS score was **0**	MD **1.93 lower** (2.35 lower to 1.51 lower)
Cecal intubation rate	2153 (7 RCTs)	⊕⊕◯◯ Low[a,b]	**RR 1.06** (1.01 to 1.11)	901 per 1,000	**54 more per 1,000** (9 more to 99 more)
Polyp detection rate (PDR)	1592 (6 RCTs)	⊕⊕◯◯ Low[a,b]	**RR 1.58** (1.23 to 2.04)	273 per 1,000	**159 more per 1,000** (63 more to 284 more)
Adenoma detection rate (ADR)	1539 (6 RCTs)	⊕⊕◯◯ Low[a,b]	**RR 1.37** (0.97 to 1.94)	271 per 1,000	**100 more per 1,000** (8 fewer to 254 more)
Cecal intubation time	1382 (5 RCTs)	⊕⊕◯◯ Low[a,b]	-	The mean cecal intubation time was **0**	MD **0.6 lower** (1.76 lower to 0.56 higher)
Withdrawal time	1382 (5 RCTs)	⊕⊕⊕◯ Moderate[a]	-	The mean withdrawal time was **0**	MD **1.15 lower** (1.56 lower to 0.73 lower)
Abdominal pain	1270 (4 RCTs)	⊕⊕◯◯ Low[a,c]	**RR 1.06** (0.72 to 1.56)	70 per 1,000	**4 more per 1,000** (20 fewer to 39 more)
Abdominal distension	1012 (3 RCTs)	⊕⊕⊕◯ Moderate[c]	**RR 0.85** (0.63 to 1.15)	156 per 1,000	**23 fewer per 1,000** (58 fewer to 23 more)
Nausea	1270 (4 RCTs)	⊕⊕◯◯ Low[a,c]	**RR 1.06** (0.88 to 1.28)	232 per 1,000	**14 more per 1,000** (28 fewer to 65 more)
Vomiting	1070 (3 RCTs)	⊕⊕◯◯ Low[a,c]	**RR 0.78** (0.41 to 1.49)	72 per 1,000	**16 fewer per 1,000** (43 fewer to 35 more)
Patient's willingness to repeat bowel preparation	1270 (4 RCTs)	⊕⊕◯◯ Low[a,b]	**RR 1.09** (0.96 to 1.23)	630 per 1,000	**57 more per 1,000** (25 fewer to 145 more)

***The risk in the intervention group** (and its 95% confidence interval) is based on the assumed risk in the comparison group and the **relative effect** of the intervention (and its 95% CI).

CI: confidence interval; **MD:** mean difference; **RR:** risk ratio

GRADE Working Group grades of evidence
High certainty: we are very confident that the true effect lies close to that of the estimate of the effect.
Moderate certainty: we are moderately confident in the effect estimate: the true effect is likely to be close to the estimate of the effect, but there is a possibility that it is substantially different.
Low certainty: our confidence in the effect estimate is limited: the true effect may be substantially different from the estimate of the effect.
Very low certainty: we have very little confidence in the effect estimate: the true effect is likely to be substantially different from the estimate of effect.

Explanations

a. One study had a large number of lost patients
b. I^2 value was large
c. Total number of events was less than 300.
